# Validity of maternal report of care-seeking for childhood illness

**DOI:** 10.7189/jogh.08.010602

**Published:** 2018-06

**Authors:** Emily D Carter, Micky Ndhlovu, Melinda Munos, Emmy Nkhama, Joanne Katz, Thomas P Eisele

**Affiliations:** 1 Institute for International Programs, Johns Hopkins Bloomberg School of Public Health, Baltimore, Maryland, USA; 2 Chainama College of Health Sciences, Lusaka, Zambia; 3 Johns Hopkins Bloomberg School of Public Health, Baltimore, Maryland, USA; 4 Center for Applied Malaria Research and Evaluation (CAMRE), Tulane University School of Public Health and Tropical Medicine, New Orleans, Louisiana, USA

## Abstract

**Background:**

Accurate data on care-seeking for child illness are needed to improve public health programs and reduce child mortality. The accuracy of maternal report of care-seeking for child illness as collected through household surveys has not been validated.

**Methods:**

A 2016 survey compared reported care-seeking against a gold-standard of health care provider documented care-seeking events among a random sample of mothers of children <5 years in Southern Province, Zambia. Enrolled children were assigned cards with unique barcodes. Seventy-five health care providers were given smartphones with a barcode reader and instructed to scan the cards of participating children seeking care at the source, generating an electronic record of the care-seeking event. Additionally, providers gave all caregivers accessing care for a child <5 years provider-specific tokens used to verify the point of care during the household survey. Reported care-seeking events were ascertained in each household using a questionnaire modeled off the Zambia Demographic and Health Survey (DHS) / Multiple Indicator Cluster Survey (MICS). The accuracy of maternal report of care-seeking behavior was estimated by comparing care-seeking events reported by mothers against provider-documented events.

**Results:**

Data were collected on 384 children with fever, diarrhea, and/or symptoms of ARI in the preceding 2 weeks. Most children sought care from government facilities or community-based agents (CBAs). We found high sensitivity (Rural: 0.91, 95% confidence interval CI 0.84-0.95; Urban: 0.98, 95% CI 0.92-0.99) and reasonable specificity (Rural: 0.71, 95% CI 0.57-0.82; Urban: 0.76, 95% CI 0.62-0.85) of maternal report of care-seeking for child illness by type of provider. Maternal report of any care-seeking and seeking care from a skilled provider had slightly higher sensitivity and specificity. Seeking care from a traditional practitioner was associated with lower odds of accurately reporting the event, while seeking care from a government provider was associated with greater odds of accurate report. The measure resulted in a slight overestimation of true care-seeking behavior in the study population.

**Conclusions:**

Maternal report is a valid measure of care-seeking for child illness in settings with high utilization of public sector providers. The study findings were limited by the low diversity in care-seeking practices for child illness and the exclusion of shops.

Despite recent reductions, under five mortality in many low- and middle-income countries (LMICs) remains high in absolute numbers. Pneumonia, diarrhea, and malaria remain the primary causes of child death in the post-neonatal period. Deaths from these three illnesses are preventable and can often be managed with simple curative interventions.

Seeking timely care from an appropriate health care provider is a necessary step in accessing correct assessment, classification, and management of child illness. A number of factors can influence decision-making around the timing and source of care for child illness [1-4]. Accurate information on the rates and patterns of care-seeking for child illness is essential for the development and direction of health programs to ensure correct management of key child illnesses.

Accurate information on the source of care is an essential component for understanding the type of care a sick child received. In most LMICs, information on treatment of suspected acute respiratory infection (ARI), malaria, and diarrhea is collected through population-based household surveys. Recent studies have shown significant limitations in the accuracy of caregiver report of management of recent child illness including receipt of a malaria test in children with fever [5] and antibiotic treatment among children with symptoms of ARI [6]. Presumption of appropriateness of reported management on the basis of illness symptoms alone is insufficient in many contexts [6,7]. Given these limitations, information on the timing and source of care may be the best predictor of correct management of child illness in many situations, particularly when coupled with timely data on the quality of care at various sources.

Information on care-seeking for child illness is commonly collected through population-based surveys, such as the Demographic and Health Survey (DHS) and Multiple Indicator Cluster Survey (MICS) that rely on maternal-report of care-seeking behaviors. Maternal report of care-seeking behavior for child illness may be subject to systematic and random error associated with autobiographical questions, including social desirability bias and telescoping [8]. Inaccuracies in maternal report of care-seeking behavior could result in misdirection of public health programming to improve the management of childhood illnesses.

Despite the importance of care-seeking behavior in the continuum of correct case management of childhood illness, there have been no previous studies assessing the validity of maternal report of care-seeking behavior for childhood illness as collected by household surveys in sub-Saharan Africa or Asia. The objective of this study was to assess the validity of maternal report of care-seeking for illness in a child <5 years old, as captured through a household survey in Southern Province, Zambia. The study aimed to estimate the sensitivity, specificity, and accuracy of maternal report of care-seeking for child illness as collected through a household survey against a gold standard of health care provider records of care-seeking events. We also assessed the association between accuracy of maternal report and socio-demographic characteristics.

## METHODS

### Study site

The study was conducted in Choma District in Southern Province, Zambia, between January 18 and March 20, 2016. The economy of Choma district is primarily agrarian, although Choma town is a growing commercial hub and provincial capital [9]. Zambia experiences three seasons, a cool dry season from May to August, a hot dry season from September to October, and a warm rainy season from November to April [10].

Child under five mortality rates in Southern Province have declined dramatically over the past 2 decades from 134 deaths per 1000 live births in 1992 to 68 deaths per 1000 live births in 2013-2014 [10]. Pneumonia, diarrhea, and malaria remain the leading causes of child under five mortality in the post-neonatal period [10]. Seasonality in child illness exists in the region, with ARI cases peaking in the dry season, diarrhea most prevalent during the rainy season, and malaria rates peaking late in the rainy season [10]. Southern Province is classified as an area with sustained malaria control resulting in malaria parasite prevalence under 10% in children under 5 years at peak transmission [11]. Reported care-seeking for child illness is high in Southern Province, with approximately 70 percent of mothers reporting they sought care for their child with fever (68.5%), diarrhea (70%), or ARI symptoms (68%) [10].

The public sector dominates health service delivery in Zambia. The government manages 90% of health facilities either directly or through service agreements with the Churches Health Association of Zambia (CHAZ). There is growing private sector involvement in urban centers [12]. Health services are free for children <5 years at all government facilities, including referral services to hospitals with presentation of a referral letter [13]. Community based health agents (CBAs) may participate in task shifting at government health centers and health posts and deploy a variable package of community-based interventions, including diagnosis and treatment of malaria and treatment of diarrhea with oral rehydration solution (ORS) in the study area [14].

### Study design, participants, and data collection

Ethical approval for the study was obtained from the Institutional Review Boards of Johns Hopkins School of Public Health and Excellence in Research Ethics and Science (ERES) Converge in Zambia.

The study included three data collection components: 1) enrollment of households with children under the age of 59 months, 2) tracking of sick child care-seeking events by health care providers, and 3) survey of participating households on care-seeking for child illness in the preceding 2 weeks. The study area was defined as the catchment population of five government health centers in and around Choma town, and stratified into urban and rural populations.

Households with children <5 years were enrolled in the study (January 18 – February 13, 2016). Households were randomly sampled from the catchment area of three rural health centers using an existing household listing created in 2014 [15]. Urban households were sampled from a census of households conducted immediately prior to the household enrollment phase. A household was defined using Zambia DHS (ZDHS) criteria [10]. Households were eligible to participate in the study if a woman of reproductive age (15-49 years) with at least one biological child <59 months resided in the household. These criteria were selected to correlate with the DHS requirements for the Women’s Questionnaire and ensure participating children were under 5 years of age at the time of the follow-up household survey. In consenting households, we conducted a brief survey on household assets, demographics, and maternal preferences in seeking care for sick children. All enrolled children <59 months were assigned a laminated card with a unique barcode number. In the event curative services were sought for a sick child, household members were instructed to present the card at the source of care. Household members were also instructed to save any ribbon given to them by a health care provider.

Health care providers were identified and recruited to track children brought to them for curative services. Care providers were defined as public, private, informal, or traditional sources of care. In each catchment area, community leaders and health workers generated a listing of care providers offering medicine or alternative treatment for sick children. Providers that treated a relatively small number of children (<5 cases per month) were excluded from the study. Ten to fifteen health providers were identified in each catchment area. Providers that agreed to participate in the study were given a smart phone with an application for reading barcodes and recording information on the time, location, and treatment given to a sick child. Providers were also given tokens, serialized Tyvek ribbons of a color corresponding to the category of health care provider (eg, blue for pharmacy, gold for traditional practitioner). Each ribbon could be traced to a specific care provider via the unique serial number. Providers were instructed to scan the barcode for any child participating in the study brought to them for care. Providers were also instructed to give a ribbon to the caregiver of any child <5 years brought to them for care, regardless of whether the child had a card. Providers were also encouraged to maintain a paper record of children brought for care. Barcode scan information was transmitted via cellular data in real-time. Where data could not be transmitted due to inconsistent cellular signal, data were manually extracted from the study phones at the end of the data collection period.

Approximately four to six weeks after enrollment, participating households were revisited and the follow-up care-seeking survey was administered (March 3 – 20, 2016). Mothers were asked a series of questions on child illness, care-seeking, and illness management identical to those asked in the ZDHS and MICS Round 5 questionnaire (see **Box 1**). Data collectors were trained to administer the questionnaire as outlined in the DHS Interviewer’s Manual [16]. Participating mothers were asked about the presence of diarrhea, fever, or suspected ARI in each of their children <5 years in the preceding two weeks. If a child experienced one or more of these illnesses, the mother was asked if any care was sought, the source of care, and treatment received. Following the completion of the series of DHS/MICS care-seeking questions, an additional questionnaire was administered to ascertain the name of the specific source of care, dates of the illness and care-seeking events, whether the barcode card was presented to the provider, and whether a ribbon was given to the caregiver at the source of care. If a caregiver received a ribbon, additional questions captured information about the color and serial number of the ribbon(s).

Box 1 **ZDHS Questions on Care-seeking for Child Illness.**

**Care-seeking for diarrhea**
Single Choice: Has [NAME] had diarrhoea in the last 2 weeks?
Single Choice: Did you seek advice or treatment for the diarrhoea from any source?
Multiple Choice: Where did you seek advice or treatment? Anywhere else? [PROBE TO IDENTIFY EACH TYPE OF SOURCE; IF UNABLE TO DETERMINE IF PUBLIC OR PRIVATE SECTOR, WRITE THE NAME OF THE PLACE.]

**Care-seeking for fever and / or ARI**
Single Choice: Has [NAME] been ill with fever at any time in the last 2 weeks?
Single Choice: Has [NAME] had an illness with cough at any time in the last 2 weeks?
Single Choice: When [NAME] had an illness with a cough, did he/she breathe faster than usual with short, rapid breaths or have difficult breathing?
Single Choice: Was the fast or difficult breathing due to a problem in the chest or to a blocked or runny nose?
Single Choice: Did you seek advice or treatment for the illness from any source?
Multiple Choice: Where did you seek advice or treatment? Anywhere else? [PROBE TO IDENTIFY EACH TYPE OF SOURCE; IF UNABLE TO DETERMINE IF PUBLIC OR PRIVATE SECTOR, WRITE THE NAME OF THE PLACE.]

**Multiple choice source(s) of care categories**
**Public Sector:**
Government hospital
Government health center/post*
Mobile hospital/clinic
Community based agent/fieldworker
Other public sector
**Private Medical Sector:**
Private hospital/clinic
Mission hospital/clinic†
Pharmacy
Private doctor
Mobile hospital/clinic
Community based agent/fieldworker
Other private sector
**Other Source:‡**
Shop
Traditional Practitioner

*MICS questionnaire records government health centers and health posts as separate categories.

†Mission hospitals / clinics are not a category in MICS questionnaire.

‡MICS “Other Source” category includes 1) Relative / Friend, 2) Shop / Market / Street, 3) Traditional Practitioner

### Primary outcome and explanatory variables

The primary study outcomes were the sensitivity, specificity, and accuracy of maternal report for three definitions of care-seeking events; 1) maternal report of the correct source of care by ZDHS provider category (see **Box 1**); 2) maternal report of any care-seeking event regardless of source of care; and 3) maternal report of care-seeking at a skilled provider. A skilled provider was defined as a source of care with clinical training in the management of one or multiple illnesses affecting children under 5. In this context, skilled providers included government, mission, and private hospitals, health centers, and health posts, private doctors, and government CBAs.

The sensitivity, specificity, and accuracy of maternal report were estimated by comparing maternal-reported care-seeking events for child illness against provider-documented care-seeking events. Provider-documented care-seeking events served as the gold standard against which the measure of maternal report was assessed. A care-seeking event was considered to be provider-documented if there was 1) record of scan of the child’s barcode by the provider, 2) report of provider-specific ribbon in household, or 3) paper record of the child in the provider’s register. Maternal report of care-seeking for child illness was ascertained from the follow-up questionnaire as described above. The interpretations of sensitivity, specificity, and accuracy are presented in Box 2. The validation study design and construction of these characteristics follow standard methodology [17].

Box 2 **Interpretation of report characteristic by care-seeking event outcome**

**Sensitivity**

Source of care: Percent of care-seeking events reported for a source of care (category) among care-seeking events that actually occurred at a source of care within the provider category

Any care-seeking: Percent of mothers who correctly reported seeking care for their sick child (from any source) among mothers of children for whom care was actually sought

Care-seeking at skilled provider: Percent of mothers who correctly reported seeking care for their sick child from a skilled provider among mothers of children for whom care was actually sought from a skilled provider

**Specificity**

Source of care: Percent of mothers who correctly reported not seeking care for their sick child among mothers of children for whom care was not sought

Any care-seeking: Percent of mothers who correctly reported not seeking care for their sick child (from any source) among mothers of children for whom care was not sought

Care-seeking at skilled provider: Percent of mothers who correctly reported not seeking care for their sick child from a skilled provider among mothers of children for whom care was not sought from a skilled provider

**Positive predictive value**

Source of care: Percent of care-seeking events that actually occurred at a source of care within a category of provider among those care-seeking events reported to have occurred at a source of care

Any care-seeking: Percent of mothers who actually sought care for a sick child among mothers who reported seeking care for a sick child

Care-seeking at skilled provider: Percent of mothers who actually sought care for a sick child from a skilled provider among mothers who reported seeking care for a sick child from a skilled provider

**Negative predictive value**

Source of care: Percent of mothers who actually did not seek care for a sick child among mothers who reported not seeking care for a sick child

Any care-seeking: Percent of mothers who actually did not seek care for a sick child among mothers who reported not seeking care for a sick child.

Care-seeking at skilled provider: Percent of mothers who actually did not seek care for a sick child from a skilled provider among mothers who reported not seeking care for a sick child from a skilled provider

**Accuracy**

Source of care: Percent of mothers whose report of source of care (category) for a sick child agreed with provider-documented care-seeking events

Any care-seeking: Percent of mothers whose report of any care-seeking for a sick child agreed with provider-documented care-seeking events

Care-seeking at killed provider: Percent of mothers whose report of seeking care for a sick child from a skilled provider agreed with documented care-seeking events among skilled providers

Characteristics of study participants were collected through the household enrollment survey. Questions on household assets, household composition, and maternal education were based on questions in the ZDHS. Household wealth was derived from a principal component analysis of household assets within each stratum (urban/rural) using an established method for estimating household wealth and divided into quintiles [18]. The number of children <5 years in the household was calculated based on the household roster. Maternal education was categorized as no or incomplete primary education, complete primary education, incomplete secondary education, and secondary complete or higher education.

### Sample size and stratification

We estimated a sample of 107 documented care-seeking events for child illness in the preceding 2 weeks was needed in both strata to estimate the sensitivity and specificity of maternal report with a precision of ±8.0%. The sample size estimate was based on a type-1 error probability of 5% (two-tailed test), an underlying sensitivity and specificity of 80%, and a design effect of 1.1 for limited clustering within the health facility catchment area due to correlation in source of care. Approximately 560 children <5 years per strata were needed to capture 107 care-seeking events assuming 27.8% of children experienced a DHS illness in the 2 weeks preceding the survey and mothers reported seeking care for 81% of those illnesses (based on data from 2011 ZDHS), 10% of care-seeking events would occur at a provider not participating in the care-seeking event tracking, and the mothers of 5% of children would be unavailable at the time of the follow-up survey. To enroll 560 children per stratum, 700 households were sampled in each stratum assuming 90% of sampled households would be available and willing to participate and a household on average had 0.88 children <5 years.

### Analysis

The study analysis was conducted in Stata 14.2 (StataCorp, College Station, Texas, USA). The primary outcomes of sensitivity, specificity, and accuracy of maternal report for the three care-seeking event measures, and associated 95% confidence intervals (CIs), were calculated using a multi-level mixed effects logistic regression model with a random intercept to account for potential clustering at the level of the health facility catchment area. In line with DHS analytical practices, we did not adjust for potential correlation of responses within a household. Prior to estimating the primary outcomes, we reclassified the reported category of source of care based on the name of the specific provider from which care was sought. For example, if a mother reported seeking care from a government hospital (when asked about the type of source), but reported the name of a government health center, the care-seeking event was reclassified as seeking care from a government health center / post. Care-seeking events reported for providers not participating in the study were excluded from the analysis. Sensitivity analyses were conducted to assess the effect of reclassification and exclusion of non-participating providers on the estimation of the primary outcomes.

Differences in accuracy of maternal report of care-seeking events by characteristics of the child, mother, household, episode, and source of care were tested through multivariable mixed effects logistic regression models allowing for a random intercept by health facility catchment area.

Using the sensitivity and specificity for each of the three maternal report indictors, we predicted the coverage of each care-seeking outcome we would expect from a household survey on maternal report of care-seeking for childhood illness modeled for a range of true care-seeking prevalence (0-100%):

Predicted Coverage = (true coverage of CS × sensitivity) + [(1 – true coverage of CS) × (1 – specificity)]

Mesured coverage was compared against true coverage to estimate the population-level validity, or inflation factor, of maternal report.

## RESULTS

A total of 335 rural household (566 children) and 469 urban households (590 children) were enrolled in the study. At follow-up, 43 households (5.3%) were unavailable to complete the survey because the participating mother(s) had moved outside of the study area or to an unknown residence for the remainder of the study period. At follow-up, 14 households withdrew consent for the follow-up survey. This resulted in a loss-to-follow-up of 7.1% of households.

Characteristics of participating children, mothers, and households are shown in **Table 1**. There was an approximately equal distribution of children by age and gender. There were slightly fewer children under one year of age due to the lag period between enrollment and the follow-up survey, which excluded neonates born within the follow-up period. The mean age of mothers was 29.6 years in the rural area and 27.1 years in the urban area. Mothers in the urban area on average had slightly higher education compared to mothers in the rural area.

**Table 1:** Characteristics of participating children, mothers, households and health care providers, by strata

	Rural			Urban		
	n	%	95% CI	n	%	95% CI
**Child age (in years):**	547			536		
0	102	18.6	15.6-22.1	102	19	15.9-22.6
1	115	21	17.8-24.6	121	22.6	19.2-26.3
2	115	21	17.8-24.6	107	20	16.8-23.6
3	109	19.9	16.8-23.5	100	18.7	15.6-22.2
4	106	19.4	16.3-22.9	106	19.8	16.6-23.4
**Child sex:**	547			536		
Female	274	50.1	45.9-54.3	276	51.5	47.3-55.7
Male	273	49.9	45.7-54.1	260	48.5	44.3-52.7
						
**Maternal age (years):**	387			450		
15-19	47	12.1	9.2-15.8	51	11.3	8.7-14.6
20-29	155	40.1	35.3-45.0	252	56.1	51.5-60.6
30-39	126	32.6	28.1-37.4	127	28.2	24.2-32.5
40-49	59	15.2	12.0-19.2	20	4.4	2.9-6.8
**Maternal education:**	387			450		
None or primary incomplete	97	25.1	21.0-29.6	81	18.2	14.9-22.0
Primary complete	118	30.5	26.1-35.3	70	15.3	12.3-18.9
Secondary incomplete	138	35.7	31.0-40.6	170	37.9	33.5-42.5
Secondary complete or higher	34	8.8	6.3-12.1	129	28.6	24.6-33.0
**Providers:**	53			22		
Government health center/post	5			2		
Government CBA/fieldworker	19			8		
Private hospital/clinic	0			4		
Pharmacy	0			5		
Traditional practitioner	29			3		

CI – confidence interval, CBA – community-based agent

All government health centers, health posts, and CBAs trained in child curative services within the study area agreed to participate in care-seeking event tracking (**Table 1**). There were no private facilities or pharmacies within the rural study area. In the urban area, four private clinics and five pharmacies participated in the study while one private clinic and one pharmacy refused to participate. In the rural area, 29 traditional or faith-based practitioners participated in the event tracking. Two churches and one traditional birth attendant in the urban area participated in the event tracking.

No mobile clinics, mission facilities, or private community-based agents were present in the study area. Choma General Hospital was located in the urban study area but excluded from the study due to low anticipated numbers of study participants seeking care at a referral facility and potential disruption caused by event tracking in a high-volume referral facility. A large number of informal shops that stocked unregulated analgesics in addition to grocery items were identified. None were included in the study because they did not receive a significant volume of care-seeking events; seeking care from a shop was reported for 1% of rural children and 5% of urban children with illness (data not shown).

Among the 1083 children included in the care-seeking survey, 34.5% of urban children and 36.4% of rural children experienced at least one illness according to DHS questions (“DHS illness”) in the 2 weeks preceding the survey (**Table 2**). Fever was the most commonly experienced symptom in both the rural and urban areas. Among those children that experienced a DHS illness, mothers reported care was sought for 78.9% of rural children and 66.5% of urban children. Reported care-seeking from more than one source was uncommon. Among those children taken for care, mothers most often reported their child was taken to a skilled provider (95.8% rural reported care-seeking events, 91.4% urban reported care-seeking events). Overall, 20 care-seeking events (3% of rural and 12% of urban reported care-seeking events) were reported with non-participating providers, including shops (11) and the general hospital (5) (Table S1 in **Online Supplementary Document(Online Supplementary Document)**). Some mothers misclassified seeking care from a government hospital or mission clinic when care had actually been sought from a government health center based on name of the facility, including 11% of rural and 1% of urban reported care-seeking events.

**Table 2:** Characteristics of reported child illness and reported care-seeking events, by strata

	Rural			Urban		
	n	%	95% CI	n	%	95% CI
	547			536		
**Proportion of children with at least one reported DHS illness**	199	36.4	32.4-40.5	185	34.5	30.6-38.6
**Reported child illness:**	199			185		
Fever	117	58.8	51.8-65.4	84	45.4	38.4-52.6
Diarrhea	23	11.6	7.8-16.8	50	27	21.1-33.9
ARI*	6	3	1.4-6.6	3	1.6	0.5-4.9
Diarrhea & fever	28	14.1	9.9-19.6	35	18.9	13.9-25.2
Diarrhea & ARI	3	1.5	0.5-4.6	0	0	-
Fever & ARI	17	8.5	5.4-13.3	10	5.4	2.9-9.8
Diarrhea, fever, & ARI	5	2.5	1.0-5.9	3	1.6	0.5-4.9
**Proportion of illnesses for which mother reported seeking care**	157	78.9	72.7-84.0	123	66.5	59.4-72.9
**Maternal reported number of sources of care among children taken for care:†**	157			123		
1	148	94.3	89.3-97.0	118	95.9	90.6-98.3
2	9	5.7	3.0-10.7	5	4.1	1.7-9.4
**Maternal reported care-seeking events:**	157			128		
Participating provider	161	97	93.0-98.7	113	88.3	81.5-92.8
Skilled provider‡	159	95.8	91.4-98.0	117	91.4	85.1-95.2

CI – confidence interval, ARI – acute respiratory infection

*ARI defined as cough with chest-related difficulty breathing.

†There was a maximum of two reported care-seeking events for a single illness.

‡Skilled provider defined as government, mission, and private hospitals, health centers, and health posts, private doctors, and government community based agents.

Maternal report of care-seeking for child illness was compared against provider-documented care-seeking events. **Table 3** presents the distribution of reported and documented (true positive), reported but undocumented (false positive), and unreported but documented (false negative) care-seeking events by provider type and strata among providers participating in event tracking. The majority of care-seeking events occurred at government health centers. CBAs accounted for a significant proportion of rural care-seeking events. A moderate number of mothers reported seeking care from public sector providers with no documentation these care-seeking events occurred. Few mothers reported seeking care in the private sector. A relatively high proportion of documented care-seeking events among traditional practitioners were unreported by mothers.

**Table 3:** Reported vs documented source of care among participating providers, by strata

	Rural			Urban		
**Provider Type**	**Reported & Documented (TP)**	**Reported, undocumented (FP)**	**Unreported, documented (FN)**	**Reported & Documented (TP)**	**Reported, undocumented (FP)**	**Unreported, documented (FN)**
**Government/public sector:**						
Government hospital	–	–	–	–	–	–
Government health center/post	112	10	5	93	17	1
Government mobile hospital/clinic	–	–	–	–	–	–
Government CBA/fieldworker	31	4	3	0	1	0
**Private sector:**						
Private hospital/clinic	0	0	0	0	0	0
Mission hospital/clinic	–	–	–	–	–	–
Pharmacy	0	0	0	0	2	1
Private doctor	–	–	–	–	–	–
Private CBA/fieldworker	–	–	–	–	–	–
**Other:**						
Shop	–	–	–	–	–	–
Traditional practitioner	4	0	7	0	0	0
Market	–	–	–	–	–	–
**Total**	147	14	15	93	20	2
**Any source**	139	13	7	94	19	1
**Skilled provider**	137	13	5	93	18	1

FP – false positive, FN – false negative, TP – true positive

Maternal report of correct source of care by provider category is presented in **Table 4**. Maternal report of correct source of care had a sensitivity, or proportion of true care-seeking events that were correctly reported, of 91.2% (95% CI 83.6-95.5%) in the rural strata and 97.9% (95% CI 92.0-99.5%) in the urban strata. The specificity of maternal report of source of care was lower at 71.4% (95% CI 57.4-82.3%) in the rural and 75.5% (95% CI 62.1-85.3%) in the urban strata. The positive predictive value (PPV), or proportion of reported care-seeking events that truly occurred, was 91.3% (95% CI 85.9-94.8%) and 82.3% (95% CI 74.1-88.3%) in the rural and urban strata respectively. The individual accuracy of maternal report of source of care, calculated as area under the receiver operating characteristic curve (AUC), was 81.1% (95% CI 74.3-87.9%) in the rural strata and 86.6% (95% CI 81.7-91.5%) in the urban strata.

**Table 4:** Accuracy of maternal report (provider category match, any provider match, skilled provider match), by strata

	Source of care (Provider category)				Any care-seeking				Care-seeking at skilled provider			
	**Rural**		**Urban**		**Rural**		**Urban**		**Rural**		**Urban**	
TP	147		93		139		94		137		93	
TP+FN	162		95		146		95		142		94	
**Sensitivity, percent (95% CI)**	91.2	83.6-95.5	97.9	92.0-99.5	95.4	89.3-98.1	98.9	92.9-99.9	96.6	90.1-98.9	98.9	92.8-99.9
TN	35		61		35		61		42		69	
TN+FP	49		81		48		80		55		87	
**Specificity, percent (95% CI)**	71.4	57.4-82.3	75.5	62.1-85.3	72.9	58.8-83.6	76.8	61.1-87.4	76.4	63.4-85.8	80.2	63.5-90.5
TP	147		93		139		94		137		93	
TP+FP	161		113		152		113		150		111	
**PPV, percent (95% CI)**	91.3	85.9-94.8	82.3	74.1-88.3	91.4	85.8-95.0	83.2	75.1-89.0	91.3	85.6-94.9	83.8	75.7-89.5
TN	35		61		35		61		42		69	
TN+FN	50		63		42		62		47		70	
**NPV, percent (95% CI)**	74.1	45.4-90.7	96.8	88.2-99.2	85.4	60.4-95.8	98.4	89.4-99.8	91.5	68.8-98.1	98.6	90.6-99.8
TP+TN	182		154		174		155		179		162	
TP+TN+FP+FN	211		176		194		175		197		181	
**Accuracy, percent (95% CI)**	86.3	80.9-90.3	87.5	81.7-91.6	89.7	84.6-93.3	88.6	82.9-92.5	90.9	86.0-94.2	89.5	84.1-93.2
**AUC, percent (95% CI)**	81.1	74.3-87.9	86.6	81.7-91.5	84.1	77.5-90.6	87.6	82.8-92.4	86.4	80.6-92.3	89.1	84.7-93.5

PPV – positive predictive value, NPV – negative predictive value, AUC – area under the curve, TP – true positive, TN – true negative, FP – false positive, FN – false negative, CI – confidence interval

Maternal report of any care-seeking event had a slightly higher sensitivity and specificity than maternal report of source of care by provider category (**Table 4**). The AUC of maternal report of any care-seeking was also higher at 84.1% (95% CI 77.5-90.6%) in the rural strata and 87.6% (95% CI 82.8-92.4%) in the urban strata.

Maternal report of care-seeking from a skilled provider had the highest sensitivity, specificity, and accuracy of the three report measures (**Table 4**). The AUC of maternal report of care-seeking from a skilled provider was high at 86.4% (95% CI 80.6-92.3%) and 89.1% (95% CI 84.7-93.5%) in the rural and urban strata, respectively.

The sensitivity, specificity, and accuracy of maternal report of care-seeking by illness type are presented in Table S2 in **Online Supplementary Document(Online Supplementary Document)**. Estimates of sensitivity and accuracy were similar across illness types, but the specificity of maternal report was lower for mothers of children with fever and/or ARI compared to children with diarrhea. However, the overall precision of illness-specific estimates was limited by low sample size when stratified by illness.

The relationship between accuracy of maternal report of care-seeking by source of care and characteristics of the child, mother, household, illness episode, and true care-seeking behavior / source of care are presented in **Table 5**. The association between these characteristics and accuracy of maternal report of any care-seeking and skilled care are presented in Table S3 in **Online Supplementary Document(Online Supplementary Document)**. There were no significant associations between accuracy of maternal report and characteristics of the child, mother, or household. Adjusting for other characteristics, mothers of children who were not taken for care (AOR = 0.04; 95% CI 0.01-0.11) had significantly lower odds of accurately reporting their care-seeking behavior (no care sought) compared to mothers who sought care from a government health center (**Table 5**). Mothers of children for whom no care was sought had lower odds of accurately reporting seeking any care and seeking skilled care (Table S3 in **Online Supplementary Document(Online Supplementary Document)**). Mothers that sought care from a traditional practitioner had significantly lower odds (AOR = 0.01; 95% CI 0.00-0.05) of accurately reporting the care-seeking event when adjusting for other characteristics (**Table 5**). Mother that only sought care from traditional practitioners also had significantly lower odds of accurately reporting any care-seeking (Table S3 in **Online Supplementary Document(Online Supplementary Document)**). Mothers of children with fever and symptoms of ARI had significantly lower odds (AOR = 0.2; 95% CI 0.05-0.88) of accurately reporting care-seeking events compared to mothers of children with fever alone. However, this association was not significant when assessing characteristics associated with accuracy of reporting any care-seeking or seeking care from a skilled provider.

**Table 5:** Characteristics associated with accuracy of maternal report of source of care by provider category

	No.	OR	95% CI	*P*-value
**Demographic characteristics**				
**Child sex:**				
Female (Ref)	200	1	–	–
Male	187	1.58	0.75-3.30	0.228
**Child age**	**387**	**1.26**	**0.95-1.67**	**0.11**
**Number of children <5 years in household**	387	1.22	0.74-2.01	0.437
**Maternal age**	**387**	**0.99**	**0.94-1.05**	**0.732**
**Maternal education:**				
None or primary incomplete (Ref)	79	1	–	–
Primary complete	82	0.84	0.26-2.65	0.761
Secondary incomplete	168	0.6	0.22-1.65	0.318
Secondary complete or higher	58	1.76	0.37-8.43	0.482
**Household wealth (quintile):**				
Poorest (Ref)	94	1	–	–
Second	71	2.71	0.77-9.55	0.122
Middle	80	0.58	0.22-1.49	0.253
Fourth	78	0.96	0.31-2.97	0.95
Highest (wealthiest)	64	0.5	0.14-1.80	0.286
**Household location:**				
Rural	211	1	–	–
Urban	176	1.34	0.55-3.29	0.524
**Illness characteristics:**				
Fever (Ref)	200	1	–	–
Diarrhea	74	2.57	0.98-6.77	0.056
ARI	9	0.68	0.12-3.95	0.664
Fever & Diarrhea	64	0.84	0.31-2.32	0.741
Diarrhea & ARI	3	1	–	–
Fever & ARI	28	0.2	0.05-0.88	0.033
Fever & ARI & diarrhea	9	0.28	0.02-3.27	0.309
**True care-seeking behavior/source of care:**				
Government health center/post (Ref)	211	1	–	–
Government CBA	34	0.24	0.05-1.15	0.074
Pharmacy	1	1	–	–
Traditional Practitioner	11	0.01	0.00-0.05	<0.001
No care sought	130	0.04	0.01-0.11	<0.001

CI – confidence interval, OR – odds ratio, CBA – community-based agent, ARI – acute respiratory infection

**Figure 1:**
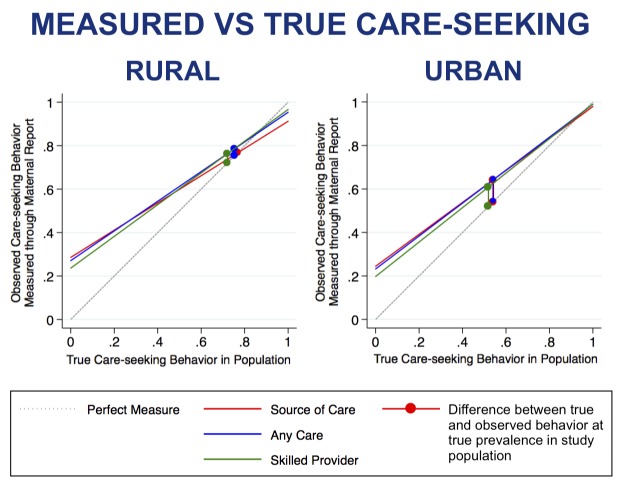
Measured vs true care-seeking behavior estimated through maternal report of care-seeking for child illness, by strata.

The modeled coverage of care-seeking by source of care ascertained from maternal report over a range of coverage scenarios demonstrates that at low coverage levels the lower specificity of maternal report would result in substantial overestimation of the proportion of sick children taken for care at the reported source of care (**Figure 1**). At high coverage levels, the high sensitivity of maternal report would result in only a slight underestimation of proportion of sick children taken for care at the reported source of care. This trend was true for estimation of any care-seeking event and seeking care from a skilled provider. In both the rural and urban strata, all three measures of maternal report had small inflation factors slightly overestimating or producing estimates of care-seeking behavior very close to the true coverage in the study population (**Table 6**). This was also true of coverage estimated by illness type (Table S4 in **Online Supplementary Document(Online Supplementary Document)**).

**Table 6:** Inflation factor for maternal report of care-seeking at true coverage in study population, by strata

	Source of care (provider category)		Any care-seeking		Care-seeking at skilled provider	
	**Rural**	**Urban**	**Rural**	**Urban**	**Rural**	**Urban**
True coverage	76.8%	54.0%	75.3%	54.3%	72.1%	51.9%
Sensitivity	91.2%	97.9%	95.4%	98.9%	96.6%	98.9%
Specificity	71.4%	75.5%	72.9%	76.8%	76.4%	80.2%
Measured coverage	76.7%	64.1%	78.5%	64.3%	76.2%	60.9%
Inflation factor	1.00	1.19	1.04	1.18	1.06	1.17

Reclassification of providers and exclusion of care-seeking events with non-participating providers did not significantly alter estimates of the sensitivity and specificity of maternal report (Tables S5-S6 in **Online Supplementary Document(Online Supplementary Document)**). Estimated sensitivity of maternal report of care-seeking by provider category in the rural area was non-significantly lower without reclassification (83%) compared to with reclassification (91%). Estimated specificity of maternal report of care-seeking by provider category in the urban area was non-significantly lower without excluding non-participating providers (64%) compared to with exclusion (76%). There was no evidence that the care-seeking event tracking methods affected maternal report of care-seeking (Table S7 in **Online Supplementary Document(Online Supplementary Document)**).

## DISCUSSION

The validity of maternal report of care-seeking for child illness was assessed by comparing maternal report against provider-documented care-seeking events. This study found high sensitivity and reasonable specificity of maternal report of care-seeking for child illness. There have been no other studies of the validity of maternal report of care-seeking for child illness in LMICs. Results from a similar study in Pune, India are forthcoming. A number of studies in high-income countries have assessed accuracy of self-reported adult health service use and found moderate to high agreement with medical records [19-23].

The observed sensitivity of maternal report of source of care was high overall but somewhat lower in rural areas, compared to the urban areas, due in large part to under-reporting of care sought from traditional practitioners. Traditional practitioners were the most common type of health provider in the rural study area, although they saw a relatively low volume of sick children. Within the study area, some local leaders and health workers openly discouraged the use of traditional practitioners, although a handful of traditional practitioners had been incorporated into the public sector as CBAs or members of local health committees or safe motherhood action groups. Concern over negative perceptions of treatment by a traditional practitioner has been cited as a potential cause of under-reporting of the use of traditional practitioners in some settings and may explain the underreporting in this setting [24,25]. Alternatively, mothers may not consider treatment by traditional practitioners to be seeking care, a point that could be clarified in the survey question administration.

The lower specificity of maternal report was driven by over-reporting of seeking care from public sector providers, including government health centers, posts, and CBAs. This over-reporting of care-seeking events in the public sector may be attributable to mothers’ expectation that researchers want to hear that care was sought for a sick child, and greater approval of treatment from a government provider, resulting in a social desirability bias [8].

The study findings were limited by the low diversity in care-seeking practices for child illness and the exclusion of shops. The majority of care-seeking events occurred in the public sector. Despite the availability of a number of private clinics and pharmacies in the urban area, very few care-seeking events were reported or documented in the private sector. Provision of free treatment for children aged under 5 in the public sector may account for low care-seeking in the private sector. The public sector is the primary source of care for child illness in many sub-Saharan African contexts [26]. However, this provider landscape may not be representative in urban areas or in other regions, limiting the generalizability of these findings.

The study cannot draw conclusions about the accuracy of maternal report of care-seeking in the private sector. As pharmacists did not provide consultations and adult formulations of medicines can be used to treat children, it was difficult for pharmacists to know when care was being sought for a child, potentially resulting in under-documentation of care-seeking events. Additionally, care-seeking from shops was not tracked because they did not meet study inclusion criteria. Although reported care-seeking from informal providers was low, absence of tracking data among shops prohibits us from assessing accuracy of maternal report as it relates to these providers.

Provider documentation of care-seeking events through barcode scans and distribution of tokens was imperfect due to issues with keeping the phone charged and accessible, caregivers failing to present the barcode card to providers, providers forgetting to distribute ribbons, and caregiver refusal or loss of ribbons. To account for these limitations, we reviewed health provider records. In the public sector and private clinics, these records were already being kept as part of routine health service tracking and were maintained separately from the study event tracking methods. This independent source of data on treatment of children strengthened the completeness of event tracking data.

While the overall accuracy of maternal report of source of care was high, we found some misclassification of source of care. Based on the originally reported source of care using the ZDHS provider categories, many mothers reported seeking care at a hospital or mission facility when the true source of care was a government health center. Misclassification was most likely due to a number of factors, including use of “hospital” as a colloquial term for health facility and changing authority from mission to government management at one health center in the study area. Misclassification was simple to identify when mothers were asked to report the name of the provider or facility from which care was sought. Inclusion of an additional question on the name of the provider, or additional unrecorded prompting to verify the category of health provider, could reduce misclassification error in household surveys but may not be feasible at scale. Additionally, some provider categories currently used in the ZDHS are non-exclusive and uninformative. CBAs in the study area ran or staffed some government health posts. However, government health posts were categorized with health centers, although the range of service and quality care offered by a CBA compared to a nurse or clinical officer could vary greatly.

This study suggests that maternal report as captured through household surveys is a valid measure of source of care for child illness in settings where utilization of public sector providers is high and diversity of care-seeking is relatively low. This finding is broadly applicable to other setting where the public sector is the primary source of care. However, there is need for additional research to assess the accuracy of maternal report of care-seeking for childhood illness in other contexts, particularly to understand report related to care-seeking in the private formal and informal sectors.
