# Supplementary material for: Validity of maternal report of care-seeking for childhood illness
Source: J Glob Health. 2018 Mar 19;8(1):010602. doi: 10.7189/jogh.08.010602 (PMC5854307; doi:10.7189/jogh.08.010602)
Supplement: Online Supplementary Document [file jogh-08-010602-s001.pdf]

Online Supplementary Document

Carter et al. Validity of maternal report of care-seeking for childhood illness

J Glob Health 2018;8:010602

Supplemental Table 1: Maternal report of source of care: original report, reclassified by specific provider name, and events reported at participating providers

Provider Type	Rural			Urban		
	Reported	Reclassified	Participating	Reported	Reclassified	Participating
**Govt / Public Sector**						
Govt hospital	12	0	-	6	5	0
Govt health center/post	105	123	122	109	110	110
Govt mobile hospital/clinic	0	0	-	0	0	-
Govt CBA/fieldworker	35	35	35	1	1	1
**Private Sector**						
Pvt hospital/clinic	0	0	-	1	1	0
Mission hospital/clinic	6	0	-	0	0	-
Pharmacy	1	1	0	2	2	2
Pvt doctor	0	0	-	0	0	-
Pvt CBA/fieldworker	1	0	-	0	0	-
**Informal**						
Shop	2	2	0	8	8	0
Traditional practitioner	4	5	4	0	0	0
Market	0	0	0	1	1	0
**TOTAL**	166	166	161	128	128	113

Supplemental Table 2. Accuracy of maternal report by reported illness type, by strata

A. Diarrhea	Source of Care (Provider Category)				Any Care-Seeking				Care-Seeking at Skilled Provider			
	Rural		Urban		Rural		Urban		Rural		Urban	
TP	44		32		40		33		39		32	
TP+FN	48		33		42		34		40		33	
**Sensitivity, percent (95% CI)**	95.1	[54.4-99.7]	97	[81.4-99.6]	95.2	[82.9-98.8]	97.1	[81.9-99.6]	97.5	[84.3-99.6]	97	[81.4-99.6]
TN	14		41		14		41		16		44	
TN+FP	17		52		16		52		18		55	
**Specificity, percent (95% CI)**	82.4	[57.3-94.2]	84.9	[43.1-97.7]	87.5	[61.4-96.9]	84.9	[43.1-97.7]	88.9	[64.8-97.2]	85.8	[46.4-97.7]
TP+TN	58		73		54		74		55		76	
TP+TN+FP+FN	65		85		58		86		58		88	
**Accuracy, percent (95% CI)**	91.9	[70.9-98.1]	89.8	[65.9-97.6]	93.1	[83.0-97.4]	89.8	[66.5-97.5]	94.8	[85.2-98.3]	90.1	[67.0-97.6]
**AUC, percent (95% CI)**	87	[76.9-97.2]	87.9	[81.6-94.3]	91.4	[82.4-100]	88	[81.7-94.3]	93.2	[85.3-100]	88.5	[82.4-94.6]

B. Fever	Source of Care (Provider Category)				Any Care-Seeking				Care-Seeking at Skilled Provider			
	Rural		Urban		Rural		Urban		Rural		Urban	
TP	132		80		123		80		122		80	
TP+FN	143		81		128		80		127		80	
**Sensitivity, percent (95% CI)**	92.3	[86.6-95.7]	98.8	[89.1-99.9]	96.3	[89.0-98.9]	100	--	96.3	[88.8-98.9]	100	--
TN	24		26		24		26		28		33	
TN+FP	34		43		34		42		38		48	
**Specificity, percent (95% CI)**	70.6	[53.4-83.4]	60.5	[45.4-73.8]	70.6	[53.4-83.4]	61.9	[46.6-75.2]	73.7	[57.6-85.2]	69.3	[52.1-82.5]
TP+TN	156		106		147		106		150		113	
TP+TN+FP+FN	177		124		162		122		165		128	
**Accuracy, percent (95% CI)**	88.1	[82.5-92.1]	85.5	[78.1-90.7]	90.7	[85.2-94.3]	86.9	[79.7-91.8]	90.9	[85.5-94.4]	88.3	[81.5-92.8]
**AUC, percent (95% CI)**	81.4	[73.4-89.5]	79.6	[72.1-87.1]	83.3	[75.4-91.3]	81	[73.5-88.4]	84.9	[77.6-92.2]	84.4	[77.7-91.0]

C. ARI	Source of Care (Provider Category)				Any Care-Seeking				Care-Seeking at Skilled Provider			
	Rural		Urban		Rural		Urban		Rural		Urban	
TP	23		11		21		11		21		11	
TP+FN	27		11		24		11		23		11	
**Sensitivity, percent (95% CI)**	85.2	[66.5-94.3]	100	--	87.6	[54.4-97.7]	100	--	93.2	[52.1-99.4]	100	--
TN	3		4		3		4		5		4	
TN+FP	5		6		5		5		7		5	
**Specificity, percent (95% CI)**	60	[20.0-90.0]	66.7	[26.8-91.6]	60	[20.0-90.0]	80	[30.9-97.3]	71.4	[32.7-92.8]	80	[30.9-97.3]
TP+TN	26		15		24		15		26		15	
TP+TN+FP+FN	32		17		29		16		30		16	
**Accuracy, percent (95% CI)**	81.3	[64.1-91.3]	88.2	[63.2-97.0]	82.6	[61.9-93.3]	93.8	[66.5-99.1]	87.4	[64.2-96.4]	93.8	[66.5-99.1]
**AUC, percent (95% CI)**	72.6	[47.6-97.5]	83.3	[62.7-100]	73.8	[48.8-98.7]	90	[70.4-100]	81.4	[62.4-100]	90	[70.4-100]

Supplemental Table 3. Characteristics associated with accuracy of maternal report of any care-seeking and seeking care from a skilled provider

Any Match	n	OR	[95% CI]	p-value
**Demographic Characteristics**				
Child Sex				
Female (Ref)	191	1	-	-
Male	178	1.56	[0.69-3.56]	0.287
Child Age	369	1.24	[0.90-1.71]	0.179
Number of children <5 years in household	369	1.41	[0.77-2.56]	0.263
Maternal Age	369	0.99	[0.93-1.05]	0.689
Maternal education				
None or primary incomplete (Ref)	77	1	-	-
Primary complete	74	1.36	[0.35-5.32]	0.658
Secondary incomplete	161	0.46	[0.15-1.39]	0.168
Secondary complete or higher	57	1.23	[0.24-6.34]	0.805
Household wealth (quintile)				
Poorest (Ref)	90	1	-	-
Second	67	3.96	[1.02-15.37]	0.047
Middle	78	0.95	[0.34-2.67]	0.927
Fourth	75	1.23	[0.35-4.32]	0.743
Highest (Wealthiest)	59	0.96	[0.22-4.09]	0.953
Household location				
Rural	194	1	-	-
Urban	175	1.63	[0.63-4.19]	0.312
**Illness Characteristics**				
Fever (Ref)	191	1	-	-
Diarrhea	73	3.53	[1.15-10.82]	0.027
ARI	9	0.55	[0.09-3.24]	0.506
Fever & Diarrhea	60	0.57	[0.19-1.71]	0.316
Diarrhea & ARI	3	1	-	-
Fever & ARI	25	0.23	[0.04-1.38]	0.107
Fever & ARI & Diarrhea	8	0.24	[0.02-3.02]	0.266
**True Care-Seeking Behavior / Source of Care**				
Public Sector Only (Ref)	229	1	-	
Traditional Only	5	0.01	[0.00-0.03]	<0.001
Public & Private	1	1	-	-
Public & Traditional	6	1	-	-
No care sought	128	0.04	[0.01-0.12]	<0.001

Skilled Provider	n	OR	[95% CI]	p-value
**Demographic Characteristics**				
Child Sex				
Female (Ref)	195	1	-	-
Male	183	1.32	[0.58-2.98]	0.511
Child Age	378	1.25	[0.91-1.72]	0.167
Number of children <5 years in household	378	1.5	[0.83-2.72]	0.184
Maternal Age	378	0.97	[0.92-1.04]	0.405
Maternal education				
None or primary incomplete (Ref)	81	1	-	-
Primary complete	74	1.28	[0.32-5.22]	0.728
Secondary incomplete	163	0.38	[0.13-1.13]	0.082
Secondary complete or higher	60	0.91	[0.18-4.64]	0.91
Household wealth (quintile)				
Poorest (Ref)	92	1	-	-
Second	68	4.75	[1.21-18.60]	0.025
Middle	79	1.16	[0.41-3.29]	0.784
Fourth	77	2.24	[0.59-8.46]	0.235
Highest (Wealthiest)	62	1.51	[0.36-6.37]	0.572
Household location				
Rural	197	1	-	-
Urban	181	2.05	[0.80-5.27]	0.135
**Illness Characteristics**				
Fever (Ref)	197	1	-	-
Diarrhea	73	2.97	[0.94-9.44]	0.064
ARI	9	0.74	[0.10-5.17]	0.758
Fever & Diarrhea	62	0.52	[0.18-1.48]	0.219
Diarrhea & ARI	3	1	-	-
Fever & ARI	26	0.25	[0.04-1.43]	0.118
Fever & ARI & Diarrhea	8	0.19	[0.02-2.44]	0.204
**True Care-Seeking Behavior / Source of Care**				
Public Sector Only (Ref)	229	1	-	-
Traditional Only	5	1	-	-
Public & Private	1	1	-	-
Public & Traditional	6	1	-	-
No care sought	137	0.04	[0.01-0.13]	<0.001

Supplemental Table 4. Inflation factor for maternal report of care-seeking at true prevalence in study population, by strata

A. Diarrhea	Source of care (Provider Category)		Any Care-seeking		Care-seeking at Skilled Provider	
	**Rural**	**Urban**	**Rural**	**Urban**	**Rural**	**Urban**
**True Prevalence**	73.8%	38.8%	72.4%	39.5%	69.0%	37.5%
**Sensitivity**	95.1%	97.0%	95.2%	97.1%	97.5%	97.0%
**Specificity**	82.4%	84.9%	87.5%	84.9%	88.9%	85.8%
**Estimated Prevalence**	74.8%	46.9%	72.4%	47.5%	70.7%	45.2%
**Inflation Factor**	1.01	1.21	1.00	1.20	1.02	1.21

B. Fever	Source of care (Provider Category)		Any Care-seeking		Care-seeking at Skilled Provider	
	**Rural**	**Urban**	**Rural**	**Urban**	**Rural**	**Urban**
**True Prevalence**	80.8%	65.3%	79.0%	65.6%	77.0%	62.5%
**Sensitivity**	92.3%	98.8%	96.3%	100.0%	96.3%	100.0%
**Specificity**	70.6%	60.5%	70.6%	61.9%	73.7%	69.3%
**Estimated Prevalence**	80.2%	78.2%	82.3%	78.7%	80.2%	74.0%
**Inflation Factor**	0.99	1.20	1.04	1.20	1.04	1.18

C. ARI	Source of care (Provider Category)		Any Care-seeking		Care-seeking at Skilled Provider	
	**Rural**	**Urban**	**Rural**	**Urban**	**Rural**	**Urban**
**True Prevalence**	84.4%	64.7%	82.8%	68.8%	76.7%	68.8%
**Sensitivity**	85.2%	100.0%	87.6%	100.0%	93.2%	100.0%
**Specificity**	60.0%	66.7%	60.0%	80.0%	71.4%	80.0%
**Estimated Prevalence**	78.1%	76.5%	79.4%	75.0%	78.1%	75.0%
**Inflation Factor**	0.93	1.18	0.96	1.09	1.02	1.09

Supplemental Table 5. Accuracy of maternal report provider category match by strata, without correcting incorrect provider classifications

	Source of Care (Provider Category)				Any Care-Seeking				Care-Seeking at Skilled Provider			
	Rural		Urban		Rural		Urban		Rural		Urban	
TP	131		92		139		94		136		93	
TP+FN	161		95		146		95		142		94	
**Sensitivity, percent (95% CI)**	83	[71.4-90.5]	96.8	[90.7-99.0]	95.4	[89.3-98.1]	98.9	[92.9-99.9]	95.8	[90.9-98.1]	98.9	[92.8-99.9]
TN	35		61		35		61		42		69	
TN+FP	49		81		48		80		55		87	
**Specificity, percent (95% CI)**	71.4	[57.4-82.3]	75.5	[62.1-85.3]	72.9	[58.8-83.6]	76.8	[61.1-87.4]	76.4	[63.4-85.8]	80.2	[63.5-90.5]
TP	131		92		139		94		136		93	
TP+FP	145		112		152		113		149		111	
**PPV, percent (95% CI)**	90.3	[84.4-94.2]	82.1	[73.9-88.2]	91.4	[85.8-95.0]	83.2	[75.1-89.0]	91.3	[85.6-94.9]	83.8	[75.7-89.5]
TN	35		61		35		61		42		69	
TN+FN	65		64		42		62		48		70	
**NPV, percent (95% CI)**	59.7	[33.4-81.4]	95.3	[86.5-98.5]	85.4	[60.4-95.8]	98.4	[89.4-99.8]	87.8	[71.1-95.5]	98.6	[90.6-99.8]
TP+TN	166		153		174		155		178		162	
TP+TN+FP+FN	210		176		194		175		197		181	
**Accuracy, percent (95% CI)**	80	[71.0-86.6]	86.9	[81.1-91.2]	89.7	[84.6-93.3]	88.6	[82.9-92.5]	90.4	[85.4-93.8]	89.5	[84.1-93.2]
**AUC, percent (95% CI)**	76.4	[69.3-83.5]	86.1	[81.0-91.1]	84.1	[77.5-90.6]	87.6	[82.8-92.4]	86.1	[80.2-92.0]	89.1	[84.7-93.5]

Supplemental Table 6. Accuracy of maternal report provider category match by strata, without excluding non-participating providers

	Source of Care (Provider Category)				Any Care-Seeking				Care-Seeking at Skilled Provider			
	Rural		Urban		Rural		Urban		Rural		Urban	
TP	147		93		139		94		137		93	
TP+FN	162		95		146		95		142		94	
**Sensitivity, percent (95% CI)**	91.2	[83.6-95.5]	97.9	[92.0-99.5]	95.4	[89.3-98.1]	98.9	[92.9-99.9]	96.6	[90.1-98.9]	98.9	[92.8-99.9]
TN	35		61		35		61		42		69	
TN+FP	54		96		53		90		57		91	
**Specificity, percent (95% CI)**	65.2	[49.0-78.5]	64.1	[48.0-77.6]	66	[52.4-77.4]	68.2	[52.8-80.5]	73.7	[60.8-83.5]	77.1	[58.5-88.9]
TP	147		93		139		94		137		93	
TP+FP	166		128		157		123		152		115	
**PPV, percent (95% CI)**	88.6	[82.8-92.6]	72.7	[64.3-79.7]	88.5	[82.5-92.7]	76.4	[68.1-83.1]	90.1	[84.3-94.0]	80.9	[72.6-87.1]
TN	35		61		35		61		42		69	
TN+FN	50		63		42		62		47		70	
**NPV, percent (95% CI)**	74.1	[45.4-90.7]	96.8	[88.2-99.2]	85.4	[60.4-95.8]	98.4	[89.4-99.8]	91.5	[68.8-98.1]	98.6	[90.6-99.8]
TP+TN	182		154		174		155		179		162	
TP+TN+FP+FN	216		191		199		185		199		185	
**Accuracy, percent (95% CI)**	84.7	[77.3-90.0]	80.6	[74.4-85.6]	87.4	[82.1-91.4]	83.8	[77.8-88.4]	89.9	[84.9-93.4]	87.6	[82.0-91.6]
**AUC, percent (95% CI)**	77.8	[71.0-84.6]	80.7	[75.7-85.8]	80.6	[74.0-87.3]	83.4	[78.4-88.3]	85.1	[79.1-91.0]	87.4	[82.8-91.9]

Supplemental Table 7. Sensitivity of maternal report provider category match by method of confirmation

	n	OR	[95% CI]	p-value
**Source of care**				
Govt health center / post (Ref)	211	1	-	-
Govt CBA	34	0.31	[0.06-1.47]	0.139
Pharmacy	1	1	[1.00-1.00]	-
Traditional practitioner	11	0.02	[0.00-0.10]	<0.001
**Confirmation Method**				
Register Only (Ref)	59	1	-	-
Ribbon	58	0.3	[0.03-2.87]	0.296
Scan	42	0.3	[0.03-3.18]	0.319
Ribbon & Scan	98	1.23	[0.11-14.15]	0.868
